# Effectiveness of Second through Sixth Line Salvage* Helicobacter pylori* Treatment: Bismuth Quadruple Therapy is Almost Always a Reasonable Choice

**DOI:** 10.1155/2016/7321574

**Published:** 2016-03-29

**Authors:** Tahir Shaikh, Carlo A. Fallone

**Affiliations:** Division of Gastroenterology, McGill University Health Center, McGill University, Montreal, QC, Canada H4A 3J1

## Abstract

*Aim.* There is a paucity of data on the efficacy of empiric* H. pylori* treatment after multiple treatment failures. The aim of this study is to examine the efficacy of empiric salvage therapy as a second through sixth line treatment.* Methods.* In this single gastroenterology center prospective study in Montreal, Canada, patients with failed* H. pylori* treatment were offered empiric salvage therapy based on the patients' previous antibiotic exposure. Enrollment occurred after 1–5 previous failed attempts and eradication determined at least 4 weeks after completion of treatment.* Results.* 205 treatments were attempted in 175 patients using 7 different regimens. Eradication was achieved in 154 attempts (PP = 81% (154/191), ITT = 75% (154/205)). Bismuth quadruple therapy (BQT) had higher eradication success (PP = 91% (102/112), ITT = 84% (102/121)) when compared to all PPI triple therapies combined (PP = 66% (49/74), absolute risk reduction (ARR): 25% (95% CI: 13–37), ITT = 62% (49/79), ARR: 22% (95% CI: 10–35), and *p* < 0.001) and when compared to levofloxacin triple therapy (PP = 66% (40/61), ARR: 26% (95% CI: 13–39), ITT = 61% (40/66), and ARR: 24% (95% CI: 10–37)). Eradication was achieved in a high proportion with BQT on attempt two (PP = 94% (67/71), ITT = 91% (67/74)), three (PP = 85% (17/20), ITT = 71% (17/24)), four (PP = 100% (11/11), ITT = 92% (11/12)), and five (PP = 86% (6/7), ITT = 75% (6/8)). Patients with previous combined bismuth and tetracycline exposure had a lower proportion of eradication compared to patients without such an exposure (PP: 60% (6/10) versus 95% (94/99), ARR: 35% (95% CI: 11–64), and *p* < 0.001; ITT: 55% (6/11) versus 90% (94/105), ARR: 35% (95% CI: 10–62), and *p* < 0.01).* Conclusions.* Salvage therapy with a bismuth quadruple regimen is superior to triple therapies and is effective for second through fifth line empirical treatment (≥85% PP, ≥70% ITT). Successful eradication is significantly lower with BQT if a similar bismuth based regimen was used in the past.

## 1. Introduction

*Helicobacter pylori (H. pylori)* is a slow growing, Gram-negative bacterium with multiple characteristics that allow it to thrive adjacent to the gastric mucosa. It is estimated that roughly fifty percent of the world's population is chronically infected with* H. pylori* [1].* H. pylori* testing has been recommended in patients with gastric MALT lymphoma, active peptic ulcer disease (PUD), and a history of PUD and patients under the age of 55 with uninvestigated dyspepsia and no alarm features, as well as in other situations [2, 3]. Despite multiple options for treatment regimens [4–8], the optimal antibiotic regimen for* H. pylori* eradication is yet to be determined. First line therapy for* H. pylori* infection includes (i) triple therapy (proton pump inhibitor (PPI), clarithromycin, and amoxicillin or metronidazole), (ii) bismuth quadruple therapy (PPI, bismuth subsalicylate, metronidazole, and tetracycline), (iii) sequential therapy (PPI and amoxicillin followed by PPI, clarithromycin, and metronidazole), or (iv) concomitant quadruple therapy (PPI, clarithromycin, amoxicillin, and metronidazole). Successful eradication with first line therapy ranges from 70 to greater than 90 percent [2].

Approximately twenty percent of all initial treatment attempts result in failure of eradication [9]. Failure of eradication may be due to primary or, in the case of prior antibiotic exposure, secondary resistance [10]. Data examining the efficacy of bismuth based therapy after failure of first line triple therapy has demonstrated an efficacy of approximately seventy percent [11–13]. Little data exists regarding the efficacy of empiric antibiotic therapy after failure of second line therapy. European guidelines suggest antibiotic susceptibility testing in the event of two treatment failures [3], although susceptibility testing is very often not available. The aim of this study is to examine the efficacy of empiric salvage therapy as a second through sixth line treatment.

## 2. Methods

### 2.1. Patient Population

In this single gastroenterology center prospective study, consecutive adult patients presenting to one of the authors (Carlo A. Fallone), a gastroenterologist in Montreal, Canada, over a four-year period (2007–2011) who had failed their first course or multiple prior courses of* H. pylori* therapy were eligible for salvage treatment. All patients were required to demonstrate persistent* H. pylori* infection either histologically or with urea breath test (UBT) prior to inclusion. Exclusion criteria included patients who were under the age of 18, refused to take further treatment, or had confirmation of* H. pylori* eradication. Patient demographics, the indication for initial testing/treatment of* H. pylori*, and exposure to the different agents used in their previous treatment attempts were recorded.

### 2.2. Study Design

**Figure 1:**
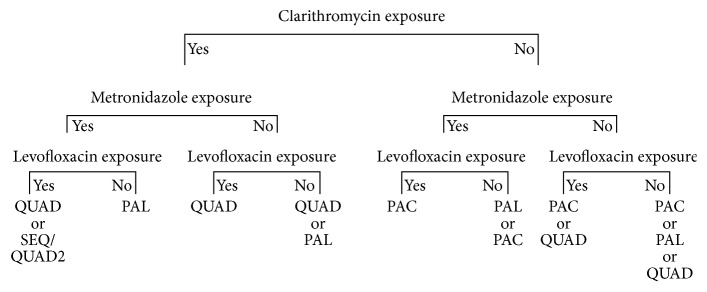
Treatment algorithm based on prior antibiotic exposure. QUAD: a twice daily proton pump inhibitor (PPI) given with four times daily metronidazole 500 mg, tetracycline 500 mg, and peptobismol 2 tablets; SEQ: a PPI and amoxicillin 1000 mg for 5 days followed by the PPI, metronidazole 500 mg, and clarithromycin 500 mg, for the subsequent 5 days, all given twice daily; QUAD2: a PPI and clarithromycin 500 mg both given twice daily along with levofloxacin 500 mg daily and peptobismol 2 tables four times a day; PAL: a PPI and amoxicillin 1000 mg, both twice daily given with levofloxacin 500 mg once daily; PAC: a PPI, amoxicillin 1000 mg and clarithromycin 500 mg, all given twice daily. In case of penicillin allergy: PAC was replaced by PMC (a PPI, metronidazole 500 mg, and clarithromycin 500 mg, all given BID) unless previous exposure to metronidazole, and PAL was replaced by PCL (a PPI and clarithromycin 500 mg, both BID given with levofloxacin 500 mg once daily) unless previous exposure to clarithromycin.

Patients with documented failed* H. pylori* treatment were offered a salvage treatment based on the previous antibiotic exposure, history of antibiotic intolerance, and without the use of antibiotic susceptibility testing. The prescribing physician (CAF) followed the following rules: (1) Antibiotics to which the patient had a known allergy were not to be used. (2) If a previous attempt included levofloxacin or clarithromycin, the salvage therapy was not to include that particular agent. (3) If the previous attempts included metronidazole, the salvage therapy was not to include metronidazole, unless rule #2 forbade any other combinations, in which case, bismuth quadruple therapy could be used. (4) The algorithm (Figure 1) demonstrates the choices available from which the physician was able to select based on previous patient intolerance to medication, dosing regiments, and so forth, in order to reflect the real world setting for this study. The antibiotic regimens are listed in Table 1 and the drug dosages are described in the associated footnote as well as below. (5) In rare cases of multiple failures where all combinations seem inappropriate or impossible because of multiple allergies, the physician could create another combination (e.g., QUAD2, described in Table 1). The listing of sequential therapy as an option on the left side of Figure 1 may seem to be inappropriate with previous clarithromycin exposure, but sequential therapy was initially felt to perform better in areas of higher clarithromycin resistance than PPI triple therapy and since no other choices are obvious in this rare setting, this choice was deemed as one that is not unreasonable.

Doses for the different treatments were as follows: A PPI was used bid with all the treatment options. Rabeprazole 20 mg was used as the PPI in all drug regimens except PAC (PPI with amoxicillin and clarithromycin), where lansoprazole 30 mg was used, as this agent is provided in a prepackaged blister pack. In addition to the PPI, for bismuth quadruple therapy (QUAD), metronidazole 500 mg qid, peptobismol 2 tablets qid, and tetracycline 500 mg qid were added. For levofloxacin triple therapy (PAL), amoxicillin 1000 mg bid and levofloxacin 500 mg daily were added to the PPI. For the other PPI triple therapies, in addition to the PPI, clarithromycin 500 mg bid was prescribed along with amoxicillin 1000 mg bid (PAC) or along with metronidazole 500 mg bid (PMC). For sequential therapy (SEQ) the PPI was given with amoxicillin 1000 mg bid for the first 5 days and then with metronidazole 500 mg bid and clarithromycin 500 mg bid for the subsequent 5 days. QUAD2 was a combination of the PPI, clarithromycin 500 mg bid, levofloxacin 500 mg daily, and peptobismol 2 tablets qid. PCL was a combination of the PPI with clarithromycin 500 bid and levofloxacin 500 mg daily. Treatment duration was 14 days for attempts 3–6 except for sequential therapy, which was 10 days. For attempt 2, duration varied from 7 days in 2 cases, 10 days in 2 cases, and 14 days in all remaining cases.

**Figure 2:**
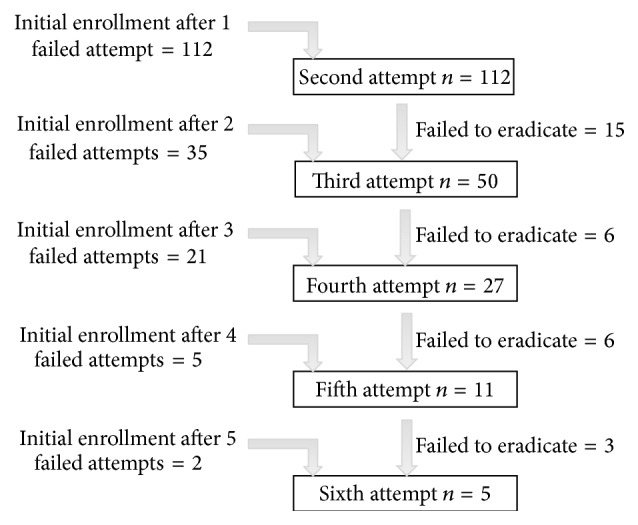
Patient enrollment. Patients referred for salvage therapy were initially enrolled after failing 1–5 attempts at* H. pylori* therapy with their referring physician. Salvage therapy was offered within this study as second to sixth attempts and those failing these attempts were offered subsequent salvage therapy within the study. The “failed to eradicate” numbers in the figure refer to those failing therapy and continuing to participate in the next treatment attempt.

Patients were initially enrolled after 1–5 previous failed attempts from referring physicians. Those failing the salvage therapy offered within the study were then offered a subsequent salvage treatment and their results were analyzed with each salvage attempt group they participated in (Figure 2).

### 2.3. Outcomes

The primary outcome measure was eradication success or failure after salvage therapy. Eradication status was confirmed at least 4 weeks after completion of the antibiotic regimen using the urea breath test (UBT) or, in cases where gastroscopy was indicated for other reasons, histological examination (Giemsa stain if negative on hematoxylin and eosin) of at least 2 gastric antral and 2 gastric body biopsies during gastroscopy. Proton pump inhibitors (PPI) were discontinued at least 2 weeks prior to any confirmatory testing. On subsequent visit or phone call by study nurse, side effects and compliance were documented. The study was approved by the institution.

### 2.4. Statistical Analysis

*H. pylori* eradication was determined by both an intention to treat (ITT, including all patients enrolled in the study and considering any patient with missing eradication data as a treatment failure) and a per protocol (PP, excluding patients for whom eradication data was unavailable) basis using data obtained from second through sixth eradication attempts (Figure 2). The 95% confidence intervals (CI) were calculated for categorical variables including the eradication proportions. Descriptive statistics for continuous variables were expressed as a mean ± standard deviation (SD). Between-group analyses were conducted by calculating the absolute risk reduction (risk difference) with 95% confidence intervals and by using the chi-square test for categorical data and Student's *t*-test for continuous variables. A *p* < 0.05 was considered to be statistically significant. Analysis was performed using SPSS software (Statistical Package for the Social Sciences version 15.0: SPSS Inc., Chicago, IL, USA).

## 3. Results

### 3.1. Study Population

One hundred and seventy-five* H. pylori* infected patients (114 female, mean age 55.4, age range 22–88) received 205 treatment attempts (Figure 2 and Table 2). Patients originated from 37 different countries, reflecting Montreal's multiethnic nature and CAF's referral basis. These included Canada (*n* = 47), Italy (*n* = 46), Greece (*n* = 9), Morocco (*n* = 6), Iran (*n* = 5), Portugal, USA, China, Algeria, Ukraine (*n* = 4 each), Trinidad (*n* = 3), and several others (*n* ≤ 2 each). The majority was Caucasian (*n* = 117, 66.9%). The indications for testing for* H. pylori* included nonulcer dyspepsia (*n* = 72, 41%), GERD (*n* = 33, 19%), a history of PUD (*n* = 28, 16%), family history of gastric cancer (*n* = 16, 9%), anemia (*n* = 15, 9%), or other (*n* = 11, 6%).

### 3.2. Treatment Regimens and Adverse Events

The first line therapy utilized by the referring physician was PPI triple therapy in 91% (159/175), with 81% versus 19% of these using a PPI and clarithromycin combined with amoxicillin versus metronidazole, respectively. Bismuth quadruple therapy was the first line in 3% (5/175) and others or unknown were used in 6% (11/175). Treatment regimens during attempts two through six were selected based on prior antibiotic exposure and patient tolerance. The most frequent salvage regimens used were bismuth quadruple and levofloxacin triple therapies (Table 1). After enrollment, four patients ultimately refused treatment, one patient died prior to initiating treatment and nine patients were lost to follow-up, so that 205 treatment attempts were used (in the 175 patients) for the ITT and 191 treatment attempts (in the 169, 164, 162, 161, and 161 patients in the second through sixth attempts, resp.) for the PP analyses.

Overall, antibiotic treatment was well tolerated with 52% (84/162) having no adverse events. Data was missing in 43 of the 205 (21%) treatment attempts. Most common adverse events were upper gastrointestinal symptoms (nausea, vomiting, and abdominal pain) at 28% (45/162), diarrhea at 5.6% (9/162), and dizziness at 4.9% (8/162). All others were found in less than 2.5% (<4/162) each and include fatigue, metallic taste, palpitations, joint pain, sore mouth, blurred vision, burning sensation, rash, and* Candida* (Table 3). The bismuth containing quadruple therapies had more overall adverse events (58/95, 61% including 38 with upper gastrointestinal symptoms, 7 with dizziness, 5 with diarrhea, 3 with fatigue, 2 with palpitations, and 1 each with metallic taste, sore mouth, and blurry vision;27 of 122 had missing data and 37 had no adverse events) than PPI triple therapies without using levofloxacin (3/12, 25% including 2 with upper gastrointestinal symptoms, and 1 with* Candida*; 1 of 13 had missing data and 9 had no adverse events), PPI triple therapies using levofloxacin (16/67, 24% including 5 with upper gastrointestinal symptoms, 4 with diarrhea, 2 with joint pain, and 1 each with metallic taste, fatigue, dizziness, burning, and rash; 15 of the 67 had missing data and 36 had no adverse events), or sequential therapy (1/3, 33% including 1 with sore mouth and 2 had no adverse events) (*p* < 0.005). Despite adverse events, only 4 of the 200 that initiated treatment did not complete therapy once it was started (2%).

### 3.3. Eradication of* H. pylori*

**Figure 3:**
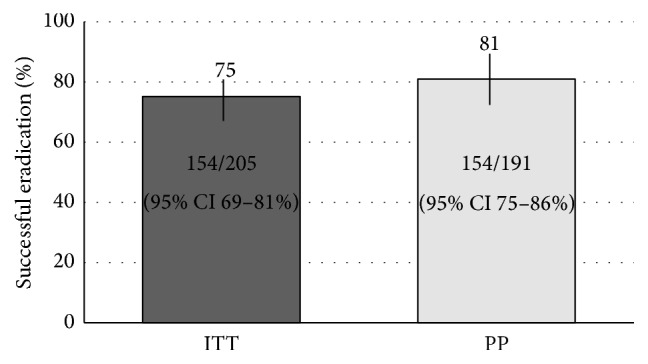
Overall* H. pylori* eradication for combined 2nd through 6th treatment attempts and including all antibiotic regimens using intention to treat (ITT) and per protocol (PP) analyses.

**Figure 4:**
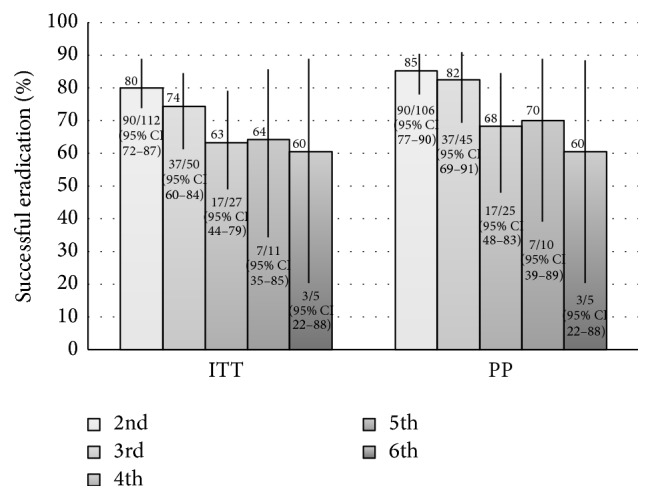
Eradication as per treatment attempt. ITT: intention to treat analysis, PP: per protocol analysis.

Overall, eradication was successful in 154 treatment attempts (PP = 81% (154/191) (95% CI: 75–86%), ITT = 75% (154/205) (95% CI: 69–81%), Figure 3). With each successive treatment attempt, the proportion of successful eradication progressively declined (Figure 4). We are unable to calculate the cumulative proportion eradication from the first attempt because we do not have the number of treated patients by the referring physicians before the first attempt failed or those referred after the second to fifth failed treatment attempts. However, in the 112 patients who underwent the second treatment attempt in our study (Figure 2), 90 succeeded (ITT: 90/112, 80%, 95% CI: 72–87%; PP: 90/106, 85%, 95% CI: 77–90%). Of the 16 confirmed failures, 15 went on to a third attempt within the study, resulting in 12 further achieving eradications, 1 confirmed failure, and 2 lost to follow-up. Hence the cumulative proportion of eradication after two subsequent attempts after initial failure was (90 + 12)/112 = 91% (ITT, 95% CI: 84–95%) or (90 + 12)/103 = 99% (PP, 95% CI: 95–100%). The one remaining confirmed failure did not go on to a subsequent attempt having passed away from unrelated causes.

**Figure 5:**
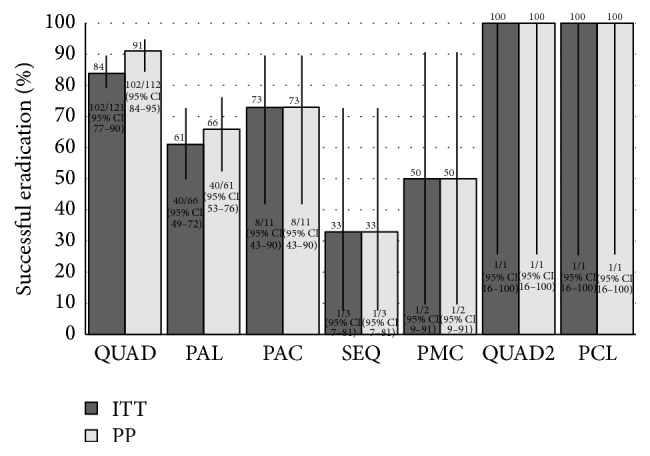
Per protocol (PP) and intention to treat (ITT) eradication of the different regimens as defined in Table 1. *p* < 0.001 (ITT or PP) for bismuth quadruple versus other combined proton pump inhibitor (PPI) triple therapies (PAL, PAC, and PMC, risk difference: 25% (PP, 95% CI: 13–37) and 22% (ITT, 95% CI: 10–35)).

When used as a salvage regimen, bismuth quadruple therapy had higher eradication success (PP = 91% (102/112) (95% CI: 84–95%), ITT = 84% (102/121) (95% CI: 77–90%)) compared to PPI triple therapies (PP = 66% (49/74) (95% CI: 55–76), ITT = 62% (49/79) (95% CI: 51–72)), with metronidazole and clarithromycin (PP and ITT = 50% (1/2) (95% CI: 9–91%)), amoxicillin and clarithromycin (PP and ITT = 73% (8/11) (95% CI: 43–90%)), or amoxicillin and levofloxacin (PP = 66% (40/61) (95% CI: 53–76%), ITT = 61% (40/66) (95% CI: 49–72%), *p* < 0.001 for both PP and ITT for bismuth quadruple versus the other 3 triple therapies combined). The absolute risk reduction (ARR) favoring bismuth quadruple therapy was 25% (PP, 95% CI: 13–37) and 22% (ITT, 95% CI: 10–35) compared to all PPI therapies and 26% (PP 95% CI: 13–39) and 24% (ITT, 95% CI: 10–37) compared to the PPI/amoxicillin/levofloxacin (PAL) combination. Those receiving PCL, SEQ, or QUAD2 (PPI with clarithromycin, levofloxacin, and bismuth) therapy were not included in the comparisons given the low numbers of subjects in these groups (Figure 5).

**Figure 6:**
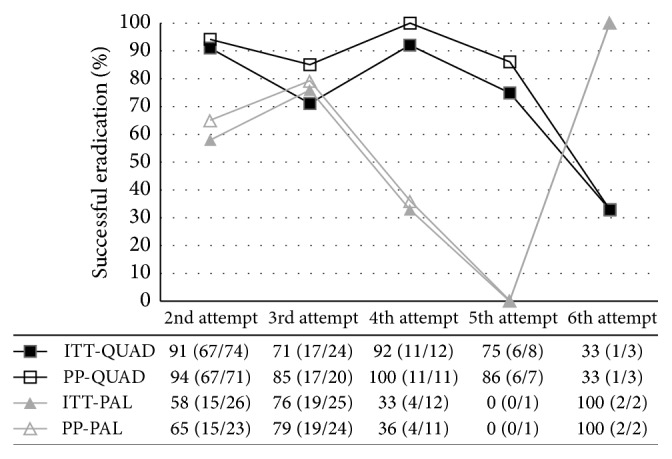
Bismuth quadruple (QUAD) and levofloxacin PPI triple therapy (PAL) eradication for each treatment attempt. ITT: intention to treat analysis, PP: per protocol analysis. Overall risk difference: 26% (PP, 95% CI: 13–39) and 24% (ITT, 95% CI: 10–37).

Bismuth quadruple therapy obtained a moderate to high proportion of successful eradication throughout most treatment attempts including attempt two (PP = 94% (67/71) (95% CI: 86–98%), ITT = 91% (67/74) (95% CI: 81–96)), three (PP = 85% (17/20) (95% CI: 63–96%), ITT = 71% (17/24) (95% CI: 51–85%)), four (PP = 100% (11/11) (95% CI: 74–100%), ITT = 92% (11/12) (95% CI: 64–98%)), and five (PP = 86% (6/7) (95% CI: 47–97%), ITT = 75% (6/8) (95% CI: 40–93%), Figure 6). Salvage therapy with PAL demonstrated variable success across attempts 2 through 6, although the numbers were small in attempts 5 and 6 for this group (Figure 6).

**Figure 7:**
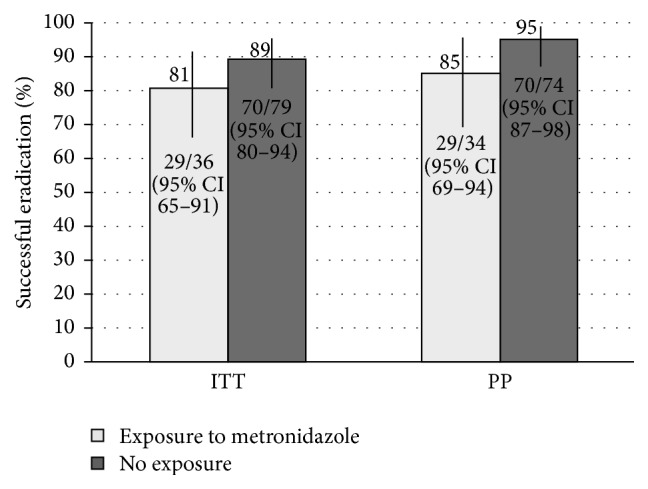
Eradication with bismuth quadruple therapy based on previous exposure to a metronidazole-containing* H. pylori* treatment. Risk difference: 10% (PP, 95% CI: −2–25) and 8% (ITT, 95% CI: −5–25).

**Figure 8:**
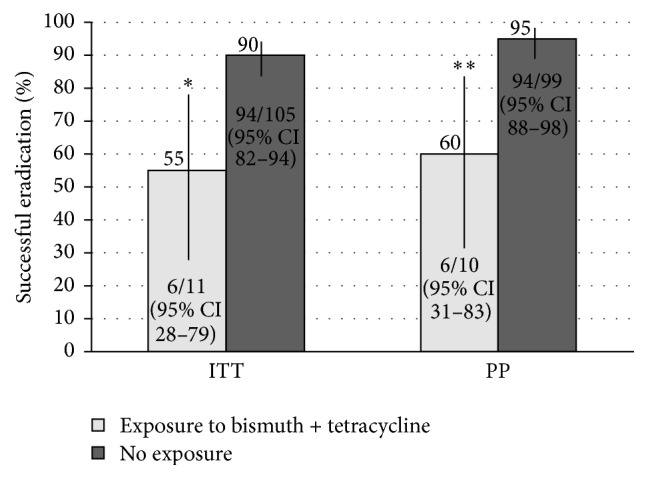
Eradication with bismuth quadruple therapy based on previous exposure to tetracycline and bismuth containing* H. pylori* treatments. ^*∗*^ *p* < 0.01, ^*∗∗*^ *p* < 0.001 for exposure versus no exposure. Risk difference: 35% (PP, 95% CI: 11–64) and 35% (ITT, 95% CI: 10–62).

The success of bismuth quadruple therapy in patients with prior exposure to* H. pylori* treatment containing metronidazole was not statistically different compared to patients without such an exposure. However, the study was not powered to detect such a difference and a trend towards reducing success with prior exposure was demonstrated (PP = 85% (29/34) (95% CI: 69–94%) versus 95% (70/74) (95% CI: 87–98%), *p* = 0.1, ARR: 10% (95% CI: −2–25), ITT = 81% (29/36) (95% CI: 65–91%) versus 89% (70/79) (95% CI: 80–94%), *p* = 0.2, ARR: 8% (95% CI: −5–25), Figure 7). Patients with a history of combined bismuth and tetracycline exposure (i.e., previous bismuth quadruple therapy attempt) had a lower proportion of successful eradication compared to patients without a history of combined bismuth and tetracycline exposure (PP: 60% (6/10) (95% CI: 31–83%) versus 95% (94/99) (95% CI: 88–98%), *p* < 0.001, ARR: 35% (95% CI: 11–64), ITT: 55% (6/11) (95% CI: 28–79%) versus 90% (94/105) (95% CI: 82–94%), *p* < 0.01, ARR: 35% (95% CI: 10–62), Figure 8).

## 4. Discussion

*H. pylori* eradication has become more difficult in recent years. In particular the success of* H. pylori* eradication with PPI triple therapy has declined significantly across the world [14]. In Canada, eradication was achieved in above 80% in the 1990s [15] but has more recently reached suboptimal levels, as low as 55% in certain communities [16, 17]. Even within the same community in Montreal, a trend of declining eradication has been demonstrated from as recently 2007 to 2011 [18]. The reduced success is not only limited to PPI triple therapy as studies have shown suboptimal eradication of* H. pylori* with newer therapies such as the sequential regimen [16, 19]. Increasing duration of therapy had been suggested as a means to improving eradication for bismuth quadruple, sequential, and PPI triple therapies [18, 20]; however this benefit seems to be disappearing with PPI triple therapy. In two Canadian studies performed in the same community, only 45% success was achieved with a 10-day course of PPI triple therapy in 2012–14 compared to 70% with a 7-day course in 2009–11 [18, 21]. Despite the longer duration, the 14-day treatment regimen's performance has also shown a decreasing trend [18].

The cause of the decreasing success of eradication treatment is felt in particular to be due to* H. pylori's* increasing antibiotic resistance to clarithromycin. In Canada, resistant strains to clarithromycin have increased from as low as 1% in the 1990s to 11% in 2003, in treatment-naïve patients, to much higher estimates especially in previously clarithromycin-exposed individuals where over 60% has been reported [16, 22–28]. Primary resistance to metronidazole, on the other hand, has remained relatively stable over time at 20–40% [24–26], explaining perhaps the reason that this treatment's success has also been stable over time [7, 29, 30].

Because of the increasing treatment failure,* H. pylori* guidelines for treatment are changing and second line or salvage treatments are becoming more challenging [3, 23, 31, 32]. Current guidelines recommend empiric bismuth based quadruple therapy as a first line treatment in areas of high clarithromycin resistance or as empirical second line salvage treatment [3]. Bismuth quadruple therapy has been demonstrated to achieve high proportion of eradication (95% PP analysis) after failure with PAC therapy [33]. Data regarding empirical bismuth based salvage therapy for third line treatment or beyond is minimal or lacking. One study evaluating a bismuth containing quadruple regimen as a third line rescue therapy after failure with PAC and PAL reported a PP and ITT eradication of 67 and 65%, respectively [34].

Our overall ITT (75%, 154/205) and PP (81%, 154/191) rate of successful eradication of* H. pylori* across all treatment attempts is comparable to the results published in the literature [34]. Our data suggests that with each failure of eradication and subsequent attempt at salvage therapy, the proportion of successful eradication declines (Figure 4). However, we were able to achieve a cumulative proportion of successful eradiation after two attempts after initial failure of 91% (ITT, 95% CI: 84–95%) or 99% (PP, 95% CI: 95–100%). When eradication success is stratified by antibiotic regimen and analyzed across all treatment attempts, bismuth based quadruple therapy appears to have a high proportion of successful eradication (ITT = 84% (102/121)) compared to PPI triple therapies combined (ITT = 62% (49/79), ARR = 22% (95% CI: 10–35)) or compared to levofloxacin triple therapy (PAL) (ITT = 61% (40/66), ARR = 24% (95% CI: 10–37), Figure 5). The relatively low success of levofloxacin triple therapy could be secondary to an already high level of background levofloxacin resistance in Canada [26]. Given that the overwhelming majority of treatment regimens were for 14 days, we cannot comment on the effect of treatment duration on success.

Bismuth based quadruple therapy maintained a consistently moderate to high proportion of successful eradication (≥85% PP, or ≥70% ITT) when used as a salvage therapy for attempts two through five (Figure 6). Successful eradication was lowest during attempt six, but the number treated in this group is too small to draw conclusions (PP = 33%, ITT = 33%, *n* = 3). Patients who had a prior exposure to bismuth quadruple therapy also demonstrated a significantly lower proportion of successful eradication compared to patients who were not exposed to bismuth quadruple therapy, although, despite previous exposure, success was still achieved in over 50% (Figure 8). Given that microbial resistance to bismuth salts has not been described, this is possibly secondary to acquired resistance due to prior metronidazole or tetracycline exposure, although tetracycline resistance is quite rare. Our data suggests that prior exposure to metronidazole did not significantly alter the efficacy of quadruple therapy as a salvage treatment, although there was a trend suggesting a reduction and the study was not powered to detect such an effect (Figure 7). In cases where there has been a history of eradication failure with a bismuth based regimen, an alternative therapy should be used if possible.

Rifabutin therapy was not used in this study because of the possibility of bone marrow suppression. However, recent studies have shown good results with this therapy and it seems to be a reasonable fourth line therapy [28, 35].

Adverse events data was available on 162 (79%) of the treatment attempts. The predominant events were gastrointestinal (nausea, vomiting, and abdominal pain (45/162, 28%)). The bismuth quadruple therapies were associated with a high proportion of adverse events but despite the high frequency of reported events, noncompletion of therapy was low (*n* = 4).

The large number of recruited patients and low dropouts is a major strength of this study. Furthermore, we obtained empirical treatment data on multiple antibiotic salvage regiments up to and including sixth line salvage treatment. All patients were consecutively enrolled and treated by a single physician. Limitations in this study include a lack of randomization. Treatment regimens were selected amongst a large number of available salvage therapies. Although the choice of therapy was based on prior antibiotic exposure, tolerability, allergy, compliance, and prior duration of therapy, selection bias (choosing one treatment versus another in certain situations) or the effect of confounding factors cannot be excluded in this nonrandomized study, perhaps affecting the treatment outcomes. Another limitation of the study is the lower number of treatment attempts with each successive attempt at salvage therapy. Finally, a large proportion of the patient population was born in Italy, potentially affecting the external validity of this study to the general North American population.

## 5. Conclusion

The proportion of successful outcome with salvage therapy declines with subsequent attempts at therapy. Salvage therapy with a bismuth based quadruple antibiotic regimen is superior to PPI triple therapy and effective for second, third, fourth, and fifth line empirical treatment (≥85% PP, ≥70% ITT). The proportion of successful eradication is significantly lower with bismuth based therapy if a similar bismuth based regimen was used in the past, although the proportion that succeeded was still above 50%.

## Figures and Tables

**Table 1:** Number of salvage treatments for each attempt.

Antibiotic regimen	Treatment attempt				
	2	3	4	5	6
QUAD (PPI + metronidazole + bismuth + tetracycline)	74	24	12	8	3
PAL (PPI + amoxicillin + levofloxacin)	26	25	12	1	2
PAC (PPI + amoxicillin + clarithromycin)	10	1			
SEQ (PPI + amoxicillin followed by PPI + metronidazole + clarithromycin)			1	2	
PMC (PPI + metronidazole + clarithromycin)	2				
QUAD2 (PPI + clarithromycin + levofloxacin + bismuth)			1		
PCL (PPI + clarithromycin + levofloxacin)			1		

PPI: proton pump inhibitor given twice daily. Doses for metronidazole were 500 mg qid for bismuth quadruple therapy (QUAD and QUAD2) and 500 mg BID for PPI triple (PMC) or sequential therapy (SEQ). Bismuth was prescribed as peptobismol 2 tablets qid, tetracycline as 500 mg qid, amoxicillin as 1000 mg bid, levofloxacin as 500 mg daily, and clarithromycin as 500 mg bid. Treatment duration was 14 days for attempts 3–6 except for sequential (SEQ), which was 10 days (5 days for part 1 and 5 for part 2). For attempt 2, duration varied from 7 days in 2 cases, 10 days in 2 cases, and 14 days in all remaining cases.

**Table 2:** Patient demographics.

	Mean, (range, ± standard deviation)
Age	55.4 (22–88, ±13.7)

	*n* (%)

Sex	
Female	114 (65)
Male	61 (35)
Race	
Caucasian	117 (67)
Asian	12 (7)
Black	12 (7)
Middle Eastern	18 (10)
Hispanic	15 (9)
Not available	1 (<1%)
Indication	
Nonulcerative dyspepsia	72 (41)
GERD	33 (19)
Peptic ulcer disease	28 (16)
Family history of gastric cancer	16 (9)
Anemia	15 (9)
Other	11 (6)

**Table 3:** Adverse effects with therapy.

Adverse effects	*n* (%)^*∗*^
None	84 (52)
Nausea, vomiting, and pain	45 (28)
Diarrhea	9 (5.6)
Dizziness	8 (4.9)
Fatigue	4 (2.4)
Metallic taste	2 (1.2)
Palpitations	2 (1.2)
Joint pain	2 (1.2)
Sore mouth	2 (1.2)
Blurry vision	1 (0.6)
Burning sensation	1 (0.6)
Rash	1 (0.6)
Candida	1 (0.6)

^*∗*^Data missing on 43 (21%) treatment attempts.
